# Aflatoxin Toxicity Reduction in Feed by Enhanced Binding to Surface-Modified Clay Additives

**DOI:** 10.3390/toxins3060551

**Published:** 2011-06-10

**Authors:** William F. Jaynes, Richard E. Zartman

**Affiliations:** Department of Plant and Soil Science, Texas Tech University, Lubbock, TX 79409, USA; Email: richard.zartman@ttu.edu

**Keywords:** aflatoxin B1, bentonites, organoclays, activated carbon, adsorption, ELISA

## Abstract

Animal feeding studies have demonstrated that clay additives, such as bentonites, can bind aflatoxins in ingested feed and reduce or eliminate the toxicity. Bentonite deposits are found throughout the world and mostly consist of expandable smectite minerals, such as montmorillonite. The surfaces of smectite minerals can be treated with organic compounds to create surface-modified clays that more readily bind some contaminants than the untreated clay. Montmorillonites treated with organic cations, such as hexadecyltrimethylammonium (HDTMA) and phenyltrimethylammonium (PTMA), more effectively remove organic contaminants, such as benzene and toluene, from water than untreated clay. Similarly, montmorillonite treated with PTMA (*K*_d_ = 24,100) retained more aflatoxin B1 (AfB1) from aqueous corn flour than untreated montmorillonite (*K*_d_ = 944). Feed additives that reduced aflatoxin toxicity in animal feeding studies adsorbed more AfB1 from aqueous corn flour than feed additives that were less effective. The organic cations HDTMA and PTMA are considered toxic and would not be suitable for clay additives used in feed or food, but other non-toxic or nutrient compounds can be used to prepare surface-modified clays. Montmorillonite (SWy) treated with choline (*K*_d_ = 13,800) and carnitine (*K*_d_ = 3960) adsorbed much more AfB1 from aqueous corn flour than the untreated clay (*K*_d_ = 944). A choline-treated clay prepared from a reduced-charge, high-charge montmorillonite (*K*_d_ = 20,100) adsorbed more AfB1 than the choline-treated high-charge montmorillonite (*K*_d_ = 1340) or the untreated montmorillonite (*K*_d_ = 293). Surface-modified clay additives prepared using low-charge smectites and nutrient or non-toxic organic compounds might be used to more effectively bind aflatoxins in contaminated feed or food and prevent toxicity.

## 1. Introduction

**Figure 1:**
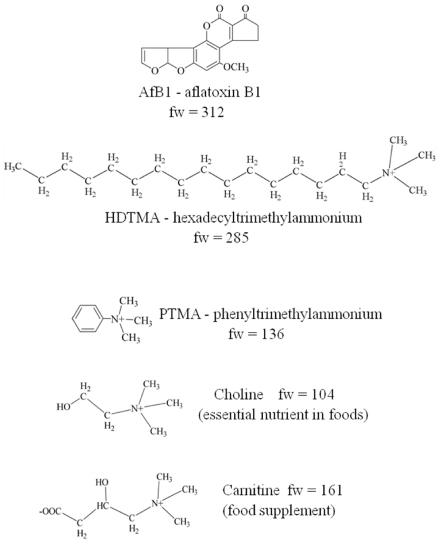
Chemical structures of aflatoxin B1, hexadecyltrimethylammonium, phenyltrimethylammonium, choline, and carnitine.

Mycotoxins are fungal metabolites toxic to vertebrates and other animal groups in low concentrations, whereas, antibiotics and phytotoxins are fungal metabolites that are toxic to bacteria and plants [1]. Mycotoxins frequently *contaminate* animal and human foods, such as peanut (*Arachis hypogaea*), corn (*Zea mays*), rye (*Secale cereale*), and cotton (*Gossypium hirsutum*) seed. In developed countries with effective regulations and food testing programs, mycotoxin contamination mostly affects animals. In developing countries, mycotoxins are a significant health risk to humans and animals. The aflatoxins are a group of mycotoxins produced by *Aspergillus* fungi that are both toxic and carcinogenic to animals and humans [2]. Aflatoxin B1 (*AfB1*) and mixtures of aflatoxin B1, G1, and M1 are proven human carcinogens [3]. Aflatoxin B1 (Figure 1) is the most toxic and abundant of the aflatoxins. An estimated 4.5 billion people living in developing countries are chronically exposed to uncontrolled amounts of aflatoxins [4].

Commercial clay additives have been used to prevent caking and improve the physical properties of animal feeds. The decreased toxicity of aflatoxins observed for contaminated animal feed mixed with clay feed additives has stimulated research on clay additives to prevent mycotoxicosis. Animal feeding studies have demonstrated that the clay additives, Novasil plus, Astra Ben 20A, Na-bentonite, zeolite, and sepiolite can effectively reduce or prevent toxicity caused by feed contaminated with *Aspergillus* mycotoxins, such as AfB1 [5,6,7,8,9,10,11,12,13,14,15]. Smectite interlayer expansion of >1.2 nm stable up to 400 °C after AfB1 treatment demonstrated that AfB1 adsorbs to interlayer clay surfaces [16]. Interlayer clay surfaces account for most of the ~800 m^2^/g surface area of smectites, such as montmorillonite. Infrared spectroscopic examination of AfB1-smectite complexes suggests that hydrogen bonds between AfB1 carbonyl groups and the hydration water of exchangeable cations in clays is the dominant bonding force under humid conditions [16]. Aflatoxin adsorption from aqueous corn and peanut meal to feed additives was consistent with animal feeding studies that used the feed additives Novasil Plus, Astra Ben 20A, sepiolite, and activated (Norit-A) carbon [17,18]. Feed additives that effectively reduced or prevented aflatoxin toxicity in feeding studies adsorbed more AfB1 from aqueous corn and peanut meal than feed additives that did not prevent toxicity.

The measured adsorption of 1 mg AfB1 to 100 mg of activated carbon (Norit-A) from aqueous media at pH 7 suggested that activated carbon might be used to prevent animal and human absorption of aflatoxins from contaminated foodstuffs [19]. Early animal feeding studies indicated that activated carbon can reduce aflatoxicosis [20,21,22]. Similarly, bentonites and activated carbon reduced excretion of aflatoxin M1 in turkey poults, milk cows, and goats in feeding studies [9,23,24]. However, animal feeding studies concluded that activated carbon does not effectively reduce aflatoxin toxicity to fed animals or is not as effective as clay additives [9,11,25]. A review of animal feeding studies did not recommend routine inclusion of activated carbon in animal diets [26]. Activated carbon is a non-specific sorbent that adsorbs essential nutrients, such as vitamin B12 and biotin, which is another drawback to use as a feed additive [27].

Clay feed additives are marketed and sold in the United States as anti-caking agents to improve the physical properties of feed because U.S. Food and Drug Administration (FDA) regulations do not permit feed additive companies to claim that feed additives can bind mycotoxins and reduce mycotoxicoses. Therefore, feed additive companies have little financial incentive to develop additives with improved mycotoxin binding. Feed additives are mixed with dry feeds and, hence, mycotoxin binding to clays must occur after ingestion. During digestion, pH, feed composition, and other factors can affect mycotoxin binding to feed additives. Mycotoxin adsorption to feed additives can sequester the toxins and limit absorption by animals or humans. Feed additives that effectively remove mycotoxins from water, however, might not prevent toxicity to animals from contaminated feed because soluble feed or digestive compounds might block mycotoxin adsorption to feed additives. 

Extensive research has shown that surface-modified clays (or organoclays) can more effectively remove organic contaminants from water than the untreated clays [28,29]. Organoclays prepared by treating smectite clays with the large organic cation, hexadecyltrimethylammonium (HDTMA, or cetyltrimethylammonium, Figure 1), effectively removed benzene and other compounds from water [30]. An HDTMA-montmorillonite removed >17 times as much ethylbenzene from water as the untreated clay [30]. The sorptive capacity of surface-modified clays prepared using large organic cations, such as HDTMA, (termed organophilic clays) is proportional to layer charge [30,31]. Contaminants are sorbed into the organic-cation-derived alkyl phase of organophilic clays by a process termed partitioning, which yields linear adsorption isotherms from water. The organic phase derived from the alkyl groups of organic cations act much like hexane in liquid-liquid contaminant extraction from water. Nonpolar organic contaminants are more soluble in hexane or the organic-cation-derived organic phase than in water. Surface-modified HDTMA-clays prepared from high-charge clays have a greater sorptive capacity for organic contaminants than those prepared using low-charge clays [30,31]. 

Organophilic clays, such as HDTMA-montmorillonite, effectively remove organic contaminants from water, but might be less effective for ingested toxins. Organophilic HDTMA- and cetylpyridinium-montmorillonites effectively sorbed zearalenone (a mycotoxin) from aqueous solution [32]. Similarly, AfB1 adsorption to sepiolite was increased by treatment with HDTMA and a biocompatible egg-yolk-derived phospholipid compound, phosphatidylcholine [33]. However, mouse uterine weight bioassay measurements later showed that HDTMA-montmorillonite does not protect mice from zearalenone toxicity [34]. Furthermore, it was suggested that the alkyl groups in organophilic clays might even assist zearalenone transport/uptake [34]. Similarly, addition of 0.4% of a proprietary organo-montmorillonite to *Fusarium* toxin-contaminated feed did not reduce the toxicity of 8.6 mg/kg deoxynivalenol and 1.2 mg/kg zearalenone to piglets [35]. Effective mycotoxin sorption from water by a feed additive does not assure reduced toxicity of ingested mycotoxins. Adsorption measurements should more accurately model the environment of ingested mycotoxins to predict whether or not a feed additive will reduce mycotoxin toxicity to animals. Clay surfaces can be modified using a great variety of compounds other than HDTMA-like alkylammonium cations.

Surface-modified clays prepared using small organic cations (termed adsorptive clays) effectively remove organic contaminants from water, but behave differently than organophilic clays. Contaminant sorption from water to adsorptive clays usually yields Langmuir-like adsorption isotherms that suggest adsorption to a surface. The contaminant sorption capacity of adsorptive clays is inversely proportional to clay layer charge [36,37]. Adsorptive clays prepared from low-charge smectites more effectively removed organic contaminants from water than adsorptive clays prepared from high-charge smectites. Contaminants apparently adsorb to the clay surfaces of adsorptive clays rather than dissolve in the alkyl-group organic phase of organophilic clays, such as HDTMA-montmorillonite. Aromatic hydrocarbon adsorption by smectite clays modified with phenyltrimethylammonium (PTMA, or trimethylphenylammonium TMPA, Figure 1), a small organic cation, was proportional to N_2_ surface area [37]. The small organic cations used to prepare adsorptive-type clays seem to act as pillars to prop open clay interlayers and allow contaminant access to interlayer clay surfaces. Contaminants must displace interlayer water to adsorb to clay surfaces. Organic cations replace strongly-hydrated inorganic cations and reduce interlayer water content in clays. Adsorptive-type surface-modified clays behave similar to untreated clays in that contaminants adsorb to interlayer clay surfaces, except that more effective contaminant adsorption might occur. 

In this study, batch AfB1 adsorption isotherms from water and from aqueous corn meal (to model ingested mycotoxins) will be measured for a variety of materials. Feed additives that have been used in animal feeding studies will be examined for comparison. Retention of AfB1 by surface-modified clays prepared from low- and high-charge reference clays will also be examined. Surface-modified clays will be prepared using HDTMA, PTMA, and non-toxic compounds. The HDTMA and PTMA organic cations are considered toxic and HDTMA- or PTMA-modified clays would not be suitable as feed ingredients. The objectives are to relate AfB1 adsorption to clay properties and identify combinations of clay charge/surface-modification compound that optimize AfB1 adsorption. Surface-modified clays, prepared using non-toxic compounds that can effectively bind aflatoxins in feed, might potentially be used in animal or human foods to prevent toxicity.

## 2. Materials and Methods

The reference clay samples, SWy-2 (SWy) and SAz-1 (SAz), were obtained from the Source Clay Repository of the Clay Minerals Society located at Purdue University (West Lafayette, IN). The SWy sample is a low-charge montmorillonite from Wyoming and SAz is a high-charge montmorillonite from Arizona. Novasil plus (a low-charge montmorillonite) is a product of Trouw Nutrition, which is a division of Englehard Corporation, Chemical Catalysts Group (600 East McDowell Road, Jackson, MS 39204). The clay samples were Na-saturated by treatment with NaCl, the <2 µm clay fractions were separated by centrifugation, and the <2 µm clay fractions were freeze-dried. Activated carbon (alkaline Norit-A decolorizing carbon) was obtained from Fisher Scientific. Dispersions of the <2 µm clays and activated carbon were prepared using an ultrasonic probe and a vortex mixer. Corn flour was obtained from a local grocery. Aflatoxin B1, rabbit anti-aflatoxin B1 antibody, aflatoxin B1-BSA conjugate, goat anti-rabbit horse-radish peroxidase (HRP) conjugate, phosphate buffered saline with 0.05% Tween 20 (PBST), and o-phenylenediamine dihydrochloride (OPD) substrate tablets were obtained from Sigma-Aldrich. Stable AfB1 stock solutions were prepared in 95% toluene/5% acetonitrile and stored in a freezer [38]. The aflatoxin stock solutions were calibrated by measuring the UV absorbance of AfB1 dissolved in methanol at 360 nm [38]. Enzyme-linked immunoassay microplates were washed and read using a Bio-Tek ELx50 plate washer and a ELx800uv (Bio-Tek Instruments, Inc.; Highland Park, Box 998, Winooski, VT) plate reader.

### 2.1. Preparation of Reduced-Charge Clay

The cation exchange capacity (CEC) and layer charge of lithium-saturated montmorillonites decrease after heat treatment. The decrease in montmorillonite CEC by lithium/thermal treatment is termed the Hofmann-Klemen effect [39]. Lithium charge reduction makes it possible to vary the CEC/layer charge of a particular clay sample and examine the effect of layer charge on other properties. A sample of <2 µm SAz-1 montmorillonite with 35% Li and 65% Na on the cation exchange sites was prepared [40]. The 0.35Li, 0.65Na-SAz sample was heated at 250 °C in a quartz glass crucible to produce about a 35% decrease in CEC/layer charge. The reduced-charge SAz clay formed, designated 0.35Li-SAz, has a CEC/layer charge comparable to SWy. 

### 2.2. Preparation of Surface-Modified Clays

Surface-modified clays were prepared by replacing the exchangeable cations in clays with organic cations. Hexadecyltrimethylammonium (HDTMA) bromide was obtained from Eastman, phenyltrimethylammonium (PTMA) chloride was obtained from Fluka, and choline chloride and carnitine hydrochloride (Figure 1) were obtained from Sigma. A weighed sample (1.63 g) of HDTMA bromide equal to the CEC (89 cmol/kg) of SWy-2 was dissolved in methanol and added to a suspension of 5 g of <2 µm SWy-2 in 500 mL of deionized water. The suspension was stirred for one hour and washed three times with deionized water by centrifugation to remove exchanged inorganic cation salts. A weighed sample (2.3 g) of PTMA chloride equal to 3 times the CEC of SWy-2 was dissolved in deionized water and added to a suspension of 5 g of <2 µm SWy-2 in 500 mL of deionized water. The suspension was stirred for one hour and washed three times with deionized water by centrifugation to remove exchanged inorganic cation salts and unadsorbed PTMA. Surface-modified clays were similarly prepared for choline and carnitine using 3 times the CEC to ensure complete cation exchange and washed with deionized water to remove exchanged inorganic cation salts and unadsorbed organic cation. The resulting SWy-HDTMA, SWy-PTMA, SWy-Choline, SWy-Carnitine, SAz-Choline, and 0.35Li-SAz-Choline clays were subsequently frozen and freeze-dried.

### 2.3. AfB1 Adsorption from Water

Batch adsorption isotherms (6 points in triplicate with 4 blanks) from water were prepared with an initial concentration of 1 µg AfB1/mL in 5 mL aqueous dispersions. The aqueous dispersions contained 10 to 180 µg of clay or activated carbon in 15 mL polypropylene centrifuge tubes. A stock solution containing 100 µg AfB1/mL in acetonitrile was prepared and aliquots of the stock solution were diluted with water and used to deliver AfB1 to isotherm solutions (50 µL stock + 0.95 mL of water = 5 µg AfB1/mL). Blanks containing only AfB1 and water were prepared and samples and blanks were thoroughly mixed using a vortex mixer. After overnight agitation on a reciprocating shaker, the tubes were centrifuged, and the supernatants were passed through 0.2-µm filters and collected in 20 mL plastic vials. The AfB1 adsorption data were fitted to the Langmuir equation and monolayer adsorption capacities (X_m_) in g AfB1/kg clay were calculated [41]. The Langmuir equation was similarly used to fit AfB1 adsorption to clay from water data [42].

### 2.4. AfB1 Retention from Aqueous Corn Flour with 60% Methanol Extraction

Aflatoxin adsorption from aqueous corn flour dispersions was used as a more applied and more conservative measure of aflatoxin retention. Batch adsorption isotherms from aquous corn meal (6 points in triplicate with 4 blanks) were prepared with an initial concentration of 1.5 µg AfB1/mL (3 µg AfB1/g corn meal) in 2 mL aqueous dispersions containing 1 to 20 mg (0.1 to 2%) clay or 5 to 100 mg (0.5 to 10%) of activated carbon. Blanks were prepared similarly using only AfB1, corn flour, and water. The 3 µg AfB1/g corn flour that was used is comparable to highly-contaminated (3000 µg aflatoxins/kg) grain. The samples were thoroughly mixed using a vortex mixer. After overnight agitation on a reciprocating shaker, 8 mL of a 60% methanol/40% 2M NaCl extracting solution were mixed with the samples using a vortex mixer. The tubes were centrifuged and the supernatants passed through 0.2-µm filters and collected in 20 mL plastic vials. Aflatoxins are more soluble in methanol than in water. The 60% methanol extraction was modified from a procedure to extract and measure aflatoxins in peanuts [43]. The AOCS method for aflatoxins in corn, cottonseed, peanut, and peanut butter similarly uses an 80% methanol/20% water extraction [44]. The modified procedure first equilibrates AfB1/corn flour/clay in water (more like the environment of ingested aflatoxins) prior to extraction with 60% methanol instead of immediate extraction [43]. The AfB1 adsorption data were fitted to a line using least squares linear regression and the slopes or adsorption coefficients (*K*_d_) calculated to use in comparing relative adsorption capacities. The *K*_d _unit (L/kg) is the ratio of adsorbed AfB1 (g AfB1/kg clay) divided by the equilibrium solution concentration (g AfB1/L). 

### 2.5. ELISA AfB1 Measurement

An enzyme-linked immunoassay (ELISA) method [43] was modified to measure AfB1 concentrations for adsorption isotherms [17]. Briefly, 96-well polystyrene microplates were coated with AfB1-BSA conjugate, washed, and saved for later use. In the first step, aflatoxin standards, sample solutions, and anti-AfB1 antibody were added to AfB1-BSA coated plates. The method is a competitive ELISA technique because AfB1 in solution (from standards or samples) competes with AfB1-BSA bound to the microplates for rabbit anti-AfB1 antibody. The AfB1 in high AfB1-concentration samples or standards binds to most of the rabbit anti-AfB1 antibody, which is subsequently washed out of the microplates. The AfB1 in low AfB1-concentration samples or standards does not bind as much of the anti-AfB1 antibody as the AfB1-BSA attached to the plates and most of the anti-AfB1 antibody is retained in the microplate. Goat anti-rabbit-HRP antibody is then added to the microplates and binds to any rabbit anti-AfB1 antibody that is attached to the microplates. Any unattached goat anti-rabbit-HRP is subsequently washed from the microplates. In the final step, OPD substrate is added to the microplates and the horse-radish peroxidase enzyme in the goat anti-rabbit-HRP that is attached to the plates catalyzes color development. The optical densities of the colored solutions that are produced are inversely proportional to AfB1 concentration. The high AfB1-concentration standards and samples are lightly-colored and the blanks are darkly-colored. The optical densities are measured using a microplate reader and AfB1 concentrations are calculated based on a plot of standard concentration *versus* optical density.

### 2.6. Basal Spacings, Organic Carbon Contents, and Specific Surface Areas

Oriented clay samples were prepared by air-drying suspensions on glass slides. The glass slides were further dried in a vacuum dessicator and stored until collection of X-ray diffraction patterns. X-ray diffraction patterns were collected for the oriented clays by scanning from 2 to 30° 2θ using CuKα radiation and a Philips diffractometer interfaced to a computer. Organic carbon contents were measured using a LECO (LECO Corporation, 3000 Lakeview Ave.; St. Joseph, MI) carbon analyzer. Clay and activated carbon samples were outgassed at 120 °C and nitrogen specific surface areas were measured by the single-point method using a Micromeritics (Micromeritics Instrument Corporation, One Micromeritics Dr.; Norcross, GA) Flowsorb II model 2300 surface area meter using a 30% N_2_/70% He carrier gas and liquid N_2_. The N_2_ surface areas of expandable clays, such as montmorillonite, are attributed to external surfaces because the interlayers collapse under the dry conditions required for measurement. Specific surface areas were also measured by ethylene glycol monoethyl ether (EGME) adsorption [45]. Samples were dried over P_2_O_5_ in a vacuum dessicator, weighed, and subsequently transferred to another vacuum dessicator with CaCl_2_-EGME solvate. Three mL of EGME were added to the (~1 g) samples and excess unadsorbed EGME was removed under vacuum until constant weight was achieved. Surface areas were calculated using the weight of adsorbed EGME. Specific surface areas measured using EGME are attributed to total surface area because EGME is absorbed into and expands the interlayers. 

## 3. Results and Discussion

### 3.1. AfB1 Adsorption from Water

**Figure 2:**
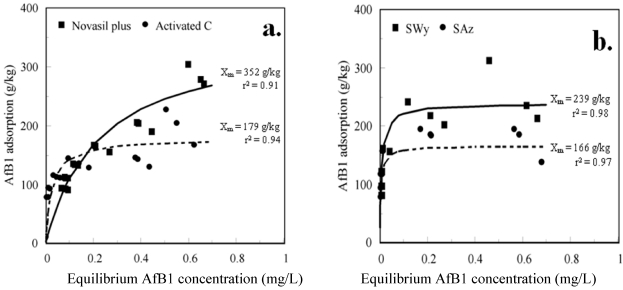
Aflatoxin B1 (AfB1) adsorption from water fitted to Langmuir equation for (**a**) Novasil plus low-charge montmorillonite and activated carbon; (**b**) low-charge SWy and high-charge SAz reference montmorillonites.

Adsorption of AfB1 to Novasil plus, activated carbon (Figure 2a), SWy, and SAz (Figure 2b) from water were comparable (~150 g/kg) at low equilibrium concentrations. The equilibrium AfB1 concentration indicates free AfB1 that could be metabolized by animals during digestion. Monolayer adsorption capacities calculated from a fit of the adsorption data to the Langmuir equation indicate some differences. The slightly greater AfB1 adsorption by Novasil plus relative to activated carbon is not comparable to the greater toxicity reduction by Novasil observed in feeding studies. The low-charge SWy montmorillonite had a somewhat greater AfB1 adsorption capacity than high-charge SAz montmorillonite. Similarly, AfB1 adsorption isotherms to Novasil from water were fitted to the Langmuir equation [42]. The Langmuir-like form of AfB1 adsorption data suggest surface adsorption, which should be limited by accessible surface area.

### 3.2. AfB1 Retention from Aqueous Corn Flour with 60% Methanol Extraction

**Figure 3:**
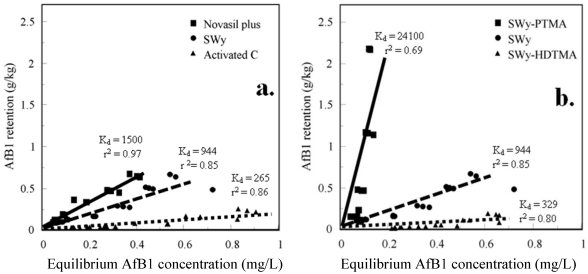
Aflatoxin B1 (AfB1) retention from aqueous corn flour after 60% methanol extraction: (**a**) Novasil plus, SWy, and activated carbon; (**b**) SWy-PTMA, SWy-HDTMA, and SWy.

Retention of AfB1 by Novasil plus and SWy from aqueous corn flour after 60% methanol extraction was much greater than activated carbon (Figure 3a), which is consistent with animal feeding studies. Novasil plus effectively prevented or reduced aflatoxicosis in feeding studies, but activated carbon was not effective or was less effective. Activated carbon has a large ~800 m^2^/g surface area (Table 1), but adsorption of soluble compounds from corn flour to external sites might block AfB1 access to much of the surface area. The maximum amounts of AfB1 retained from aqueous corn flour (~1 g/kg) were about 200 times less than from water, but the relative amounts retained were consistent with animal feeding studies. Aflatoxins are more soluble in methanol than in water and methanol might desorb weakly-adsorbed AfB1. Novasil plus and SWy are both low-charge montmorillonites and retained comparable amounts of AfB1. Feed components can interfere with aflatoxin adsorption to feed additives. Aflatoxin retention from aqueous corn flour or feed can better model aflatoxin adsorption to feed additives in contaminated feed during digestion by animals than simple adsorption from water. Simulated gastrointestinal fluids might be added to corn flour in future research to further improve the correlation between aflatoxin adsorption tests and animal feeding studies.

**Table 1:** Basal spacings (d001), organic carbon content (OC), nitrogen (N_2_) surface areas, and ethylene glycol monoethyl ether (EGME) surface areas of clay samples.

Sample	d001 (nm)	OC (%)	N_2 _(m^2^/g)	EGME (m^2^/g)
Na-Novasil plus	1.26	0.14	70	721
Na-SWy-2	1.23	0.06	29	691
SWy-HDTMA	1.82	14.60	7	216
SWy-PTMA	1.48	8.16	59	406
SWy-Choline	1.41	4.02	140	273
SWy-Carnitine	1.39	5.10	32	202
Na-SAz-1	1.26	0.07	93	735
SAz-Choline	1.41	5.68	58	275
0.35LiSAz-Choline	1.42	4.11	213	425
Norit-A activated carbon	NA	78.40	800	881

NA = not applicable.

### 3.3. AfB1 Retention by HDTMA- and PTMA-Surface-Modified Clays

**Figure 4:**
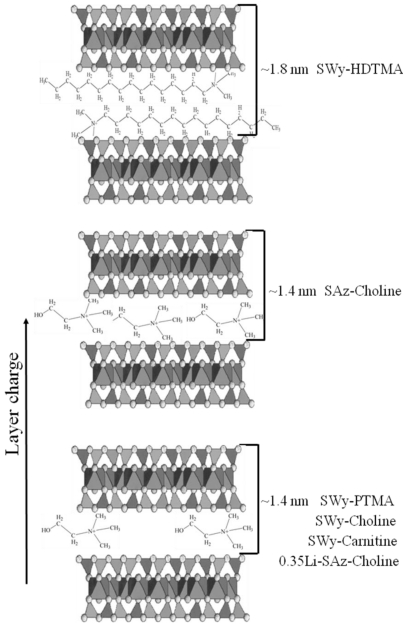
Interlayer representation of surface-modified clays.

Retention of AfB1 by untreated SWy and by the SWy-PTMA and SWy-HDTMA surface-modified clays differed greatly (Figure 3b). Compared to untreated SWy, SWy-PTMA retained much more AfB1 (*K*_d_ = 24,100) and SWy-HDTMA (*K*_d_ = 265) retained much less than SWy (*K*_d_ = 944). This suggests that SWy-HDTMA would not be an effective feed additive to prevent aflatoxicosis. An HDTMA-treated montmorillonite effectively sorbed zearalenone from water, but did not reduce zearalenone toxicity to mice [32,34]. No animal feeding studies to determine the effect of HDTMA-montmorillonite on aflatoxin toxicity have been identified. Interlayer HDTMA might limit N_2_ and AfB1 access to interlayer clay surfaces. The N_2_ surface area and basal spacing (Table 1) of SWy-PTMA (59 m^2^/g, 1.48 nm) were greater than untreated SWy (29 m^2^/g, 1.23 nm), but the SWy-HDTMA (7 m^2^/g, 1.82 nm) surface area was less and the basal spacing was greater. The PTMA in SWy-PTMA acts to prop open interlayers and the pore space around PTMA cations permits N_2_ and AfB1 access to interlayer clay surfaces. A likely arrangement of HDTMA and PTMA organic cations in SWy-HDTMA and SWy-PTMA interlayers is illustrated in Figure 4 based on X-ray diffraction basal spacings and measured N_2_ surface areas (Table 1). The basal spacings indicate the interlayer expansion of the clays after treatment with cationic organic compounds and the organic carbon contents reveal how much organic compound was retained by the clays (Table 1). The basal spacings of Novasil plus, SWy, and SAz should be ~1 nm during N_2_ surface area measurements because degassing and heating at 120 °C collapse the interlayers. In contrast, organic interlayer cations (PTMA, HDTMA, choline, and carnitine) prevent interlayer collapse during N_2_ surface area measurements. The N_2_ surface areas of SWy-TMPA, SWy-Choline, and 0.35LiSAz-Choline were greater than the untreated clays and indicate N_2_ access to interlayer surfaces. In contrast, the N_2_ surface area of SWy-HDTMA was less than the untreated clay. The EGME surface areas of the untreated clays were approximately 700 m^2^/g, which is comparable to the total surface area of ~800 m^2^/g possible for smectites. Treatment with the organic cations decreased the EGME surface areas of the surface-modified clays and might be expected to reduce maximum AfB1 adsorption from water. For ingested toxins, however, the amount of AfB1 that a feed additive can strongly adsorb from feed is probably more important than absolute adsorption capacity. The activated carbon ~800 m^2^/g surface areas for both N_2_ and EGME were similar because unlike smectites, activated carbon has no interlayers that can collapse and limit N_2_ access during N_2_ surface area measurements. 

### 3.4. AfB1 Retention by Nutrient-Compound Surface-Modified Clays

**Figure 5:**
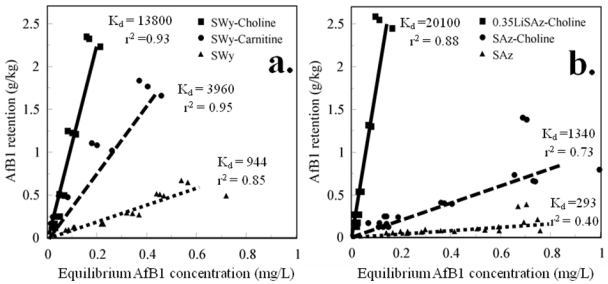
Aflatoxin B1 (AfB1) retention from aqueous corn flour after 60% methanol extraction: (**a**) SWy-Choline, SWy-Carnitine, SWy; (**b**) 0.35LiSAz-Choline, SAz-Choline, SAz.

Samples of SWy treated with the nutrient compounds, choline and carnitine, retained more AfB1 from corn flour than untreated SWy (Figure 5a). The choline-treated, high-charge, SAz montmorillonite (SAz-Choline) retained more AfB1 than untreated SAz (Figure 5b) and comparable amounts as Novasil plus (Figure 3a), but much less than SWy-Choline or SWy-PTMA. The reduced-charge 0.35LiSAz-Choline montmorillonite retained amounts of AfB1 comparable to SWy-PTMA and SWy-Choline and considerably more than SAz-Choline. Lithium charge reduction would likely be impractical for the production of low-charge feed additives, but demonstrates that layer charge is an important factor in selecting clay additives to bind aflatoxins in feed. Naturally-occurring, low-charge montmorillonites or other low-charge smectites would be more effective feed additives than higher-charge clays. Treatment of low-charge clays with small, non-toxic organic compounds, such as choline and carnitine, produce surface-modified clays (or organoclays) that might be used as feed additives to more effectively bind aflatoxins in feed and prevent toxicity. Preliminary research has shown that other small compounds, such as lysine, aspartame, and phenylalanine, similarly yield surface-modified clays that more readily bind AfB1 than untreated clay. Animal feeding studies would be needed to prove that surface-modified clays, such as SWy-Choline, can reduce the toxicity of ingested aflatoxins as effectively as the adsorption experiments suggest. 

## 4. Conclusions

Aflatoxin B1 (AfB1) adsorption from water was comparable for Novasil plus, activated carbon, and other clays. Animal feeding studies, however, have shown that Novasil plus effectively prevents aflatoxin toxicity, but activated carbon is much less effective. In contrast, AfB1 retention from aqueous corn flour was consistent with animal feeding studies. Novasil plus retained much more AfB1 from aqueous corn flour than activated carbon. A low-charge montmorillonite modified with the small phenyltrimethylammonium organic cation (SWy-PTMA) retained much more AfB1 from aqueous corn flour than the untreated clay. The PTMA cation is considered toxic and would not be acceptable for use in feed additives, but choline and carnitine are small nutrient compounds that would be suitable in feed additives. Low-charge montmorillonites modified with choline and carnitine (SWy-Choline, SWy-Carnitine) retained much more AfB1 from aqueous corn flour than Novasil plus and retained comparable amounts as SWy-PTMA. Low-charge clays modified with choline, carnitine, or other small nutrient or non-toxic compounds might be used in animal feed or human food to reduce aflatoxin toxicity.
